# Case of Total Blindness, Occurring during the Administration of Large Doses of the Sulphate of Quinine

**Published:** 1845-11

**Authors:** Henry C. Lewis

**Affiliations:** Yazoo City, Mississippi; Louisville


Art. III.—*Case of total Blindness, occurring during the administration of large doses of the Sulphate of Quinine.* Reported by Henry C. Lewis, of Yazoo City, Miss.

On the 23d of April, 1845, I was directed by my preceptor, Dr. W. Dorsey, to visit Mrs. M., aged about 65 years, residing near Yazoo City. On my first visit I found her laboring under gastro-enteritis, with the following symptoms; pulse quick, small, and not easily compressed, excessive pain in the abdomen increased by pressure, vomiting, a sensation of burning in the stomach on the admission of drinks; watery discharges nearly colorless, attended with tormiina, skin dry and hot, tongue, in the middle, coated with brown fur, edges clean and very red, great debility; patient lying on her back with lower extremities slightly drawn up; great thirst, but inability, in consequence of the vomiting to retain anything on the stomach; patient being in indigent circumstance had allowed the disease to proceed several days without sending for a physician. Prescribed cupping over the whole surface of the abdomen, mustard plaster to the same, and after it had produced its rubefacient effect, the application of a warm mush poultice, to be removed when cold; calomel, ipecac., and morphia in small doses; the only drink to be taken, slippery-elm infusion, a tablespoonful at a time.

24th. Found her somewhat relieved; discharges from the bowels ceased; tongue still coated in the middle, edges red; pain in the abdomen, though not as severe as the day before; vomiting continues at intervals. Prescribed cupping, renewal of the poultice, also of the mustard whenever vomiting occurred.

℞ Ol. Ricini. ℥ij.
Tinct. opii min. xl.
M.

One-half to be taken in the morning, and the balance in the evening if no motion had been produced; calomel and ipecac. still to be given at intervals, also slippery-elm infusion as a drink.

25th. Found the patient no better; not much heat or pain of the abdomen, vomiting increased; pulse very small and weak; extremities cold and moist; tongue somewhat cleaner; great debility; the oil had operated, producing dark thin discharges. Prescribed sulphate of quinine, one grain every hour, wine whey as much as the stomach would bear, mustard to the abdomen, to be applied very frequently. Saw her twice more to-day; continued the same treatment.

26th. Patient worse; pulse scarcely perceptible; hands and forehead bedewed with cold clammy perspiration; tongue cleaner, but tremulous; great debility; inability to retain any thing long in the stomach; no pain in the stomach or bowels on pressure; has had one or two passages of more consistency and of a bilious character; has the usual symptoms resulting from the continued administration of quinine, viz: deafness and roaring in the head; passed a restless night, with slight delirium. Prescribed sulphate of quinine, ℨij., to be divided into 24 powders, one to be given every hour until reaction should take place; wine whey, mustard to the stomach previous to the administration of the whey and quinine, as during the detraction of the irritability of the stomach to the skin, the medicine would be more likely to be retained; mint plaster to the same.

Saw her again in the evening; situation nearly the same; directed the continuation of the quinine during the night, and as the wine whey was unpalatable, directed simple Madeira wine and sugar; mustard still to be applied if vomiting should occur.

27th. Found her much better this morning; pulse improved; skin moist and soft to the touch; tongue cleaner; alvine discharges bilious, and of a proper consistency; vomiting allayed; hearing but slightly affected, but was surprised to find her totally blind. I thought, at first, that it was only imaginary, but satisfied myself by experiment that she was really blind, though her eyes presented no morbid appearance with the exception of very slight dilatation of the pupils. She complained of no pain in them, has had none, could see perfectly on the evening previous; during the night slept a little, and on waking complained to her children of a dark cloud being before her eyes. I did not attempt to do anything for her sight, but addressed my treatment to her original disease; from her situation it would have been extremely hazardous to have discontinued the tonics and stimulants, and as I knew of nothing that would be so efficient as the quinine, I resolved to continue it, but in smaller doses, as I feared that the blindness had been caused by the large quantity administered. I therefore gave three grains every three hours, continued the mint plaster to the abdomen, and the Madeira wine, with sulph. morphia ½ grain to be divided into two powders, one to be given at bed time, and the other in an hour if sleep was not produced; with directions not to disturb her for the purpose of giving the quinine in case she slept.

28th.—2 o'clock, A.M. Was sent for in great haste, and was informed that the patient had alvine discharges every few minutes; but on my arrival I was agreeably surprised to find her resting comfortably in bed. I prescribed the following:

℞ Cretæ ppt., v grs.

Acet. plumb. i. gr.

Sulph. morph., ¼ gr.

Mix.

A portion to be taken at once, also an enema of thirty drops of laudanum, and a gill of starch water to be administered. Remained with her during the night, and until eight o'clock in the morning. She had no more passages during the night. Quinine to be given in the same doses as during the day previous; wine to be allowed, and any light diet that she fancied.

3 o'clock P.M. Found on my visit that the messenger on the night previous had misinformed me, and that I had not been particular enough in my enquiries of the family; that the patient, instead of having been purged every few minutes had only made ineffectual efforts to stool, and that my prescription of astringents had been unnecessary, but it was then too late to correct it, and the patient seemed to be much improved—I will not undertake to say by the prescription; but she was doing very well; her pulse was good; skin moist and soft; no vomiting; appetite better than it had been at any time during her illness; but she still remained blind. I ordered the quinine and wine to be continued, also directed enemas of a gill of starch water, with a teaspoonful of pulv. colomba mixed in it, and ¼ grain sulph. morph. at night.

29th. Still improving; same treatment as the day before to be continued; sight no better.

30th. Patient in same situation, treatment the same.

May 1st. Still improving; continue the treatment.

2d. Ceased to visit her to-day.

14th. Have applied blisters behind the ears frequently, but her sight is still extinct; ordered the irritation from the blisters to be continued.

June 3d. Have not seen my patient lately, but understood from her son that her sight is gradually returning with her improving health.

September 16th. Heard from the patient to day; she does not see well yet; on the occurrence of any slight indisposition her sight fails her almost totally, and remains thus until her health is restored, when it returns imperfectly.

*Remarks.*—The above case I conceive to be one of peculiar interest to the profession. The question naturally arises, was the blindness occasioned by the quinine, or by the conjoined influence of age, debility, and disease? The question is a difficult one to answer; much might be said on both sides. The fact is well known that quinine has been given in much larger doses, even to the extent of an ounce in twelve hours, as it was by my preceptor, Dr. Dorsey, in several cases of congestive fever (so termed) with the best results, and without producing a single bad effect upon the system of the patient. But we must admit that a medicine which acts so frequently upon the sense of hearing, may also, when assisted by idiosyncrasy or other fortuitous circumstances, act also upon the visual organs. In fact, this ought to occur more frequently than it does, as there is great analogy between the auditory and optic nerves, they both, according to Meckel, having double origins. That it has a sort of elective affinity for the auditory organs rather than for other parts, seems to be established by the general experience of the profession. That the blindness in this case may have been caused by the conjoined influence of disease, age, and debility is also not improbable, since any one of these causes is sufficient to produce the result; but it was not debility, as it did not occur until her strength was improving; the same objection rests against its being disease; and if it was age, why did it not occur before? Doubtless it was due to several causes, quinine being the predominant one, but rendered operative by some iodiosyncrasy in the patient.

Louisville, October, 1845.

